# Combined effect of gallic acid and zinc ferrite nanoparticles on wheat growth and yield under salinity stress

**DOI:** 10.1038/s41598-024-63175-9

**Published:** 2024-06-04

**Authors:** Qingqin Shao, Lantian Ren, Musarrat Ramzan, Muhammad Baqir Hussain, Rahul Datta, Hesham S. Almoallim, Mohammad Javed Ansari, Abdullah Ehsan

**Affiliations:** 1 College of Agriculture/Anhui Intelligent Crop Planting and Processing Technology Engineering Research Center, Anhui Science and Technology University, Fengyang, 233100 Anhui China; 2 Department of Botany, The Islamia University of Bahawalpur, Bahawalpur, Pakistan; 3 Department of Soil and Environmental Sciences, MNS University of Agriculture, Multan, 60000 Punjab Pakistan; 4 Department of Geology and Pedology, Faculty of Forestry and Wood Technology, Mendel University in Brno, Zemedelska 1, 61300 Brno, Czech Republic; 5 Department of Oral and Maxillofacial Surgery, College of Dentistry, King Saud University, PO Box-60169, 11545 Riyadh, Saudi Arabia; 6 Department of Botany, Hindu College Moradabad (Mahatma Jyotiba Phule Rohilkhand University Bareilly), Moradabad, India; 7 Department of Soil Science, Faculty of Agricultural Sciences and Technology, Bahauddin Zakariya University, Multan, Punjab Pakistan

**Keywords:** Gallic acid, Chlorophyll content, Zinc ferrite nanoparticles, Antioxidant, Growth attributes, Plant sciences, Plant stress responses, Abiotic, Salt

## Abstract

Salinity stress significantly impacts crops, disrupting their water balance and nutrient uptake, reducing growth, yield, and overall plant health. High salinity in soil can adversely affect plants by disrupting their water balance. Excessive salt levels can lead to dehydration, hinder nutrient absorption, and damage plant cells, ultimately impairing growth and reducing crop yields. Gallic acid (GA) and zinc ferrite (ZnFNP) can effectively overcome this problem. GA can promote root growth, boost photosynthesis, and help plants absorb nutrients efficiently. However, their combined application as an amendment against drought still needs scientific justification. Zinc ferrite nanoparticles possess many beneficial properties for soil remediation and medical applications. That’s why the current study used a combination of GA and ZnFNP as amendments to wheat. There were 4 treatments, i.e., 0, 10 µM GA, 15 μM GA, and 20 µM GA, without and with 5 μM ZnFNP applied in 4 replications following a completely randomized design. Results exhibited that 20 µM GA + 5 μM ZnFNP caused significant improvement in wheat shoot length (28.62%), shoot fresh weight (16.52%), shoot dry weight (11.38%), root length (3.64%), root fresh weight (14.72%), and root dry weight (9.71%) in contrast to the control. Significant enrichment in wheat chlorophyll a (19.76%), chlorophyll b (25.16%), total chlorophyll (21.35%), photosynthetic rate (12.72%), transpiration rate (10.09%), and stomatal conductance (15.25%) over the control validate the potential of 20 µM GA + 5 μM ZnFNP. Furthermore, improvement in N, P, and K concentration in grain and shoot verified the effective functioning of 20 µM GA + 5 μM ZnFNP compared to control. In conclusion, 20 µM GA + 5 μM ZnFNP can potentially improve the growth, chlorophyll contents and gas exchange attributes of wheat cultivated in salinity stress. More investigations are suggested to declare 20 µM GA + 5 μM ZnFNP as the best amendment for alleviating salinity stress in different cereal crops.

## Introduction

A major global issue to agriculture is soil salinity, which is the second most important factor contributing to land degradation after soil erosion^1–5^. Approximately 1 billion hectares of salinity, or 7% of the Earth's surface, results in the daily loss of 2000 hectares of arable land, greatly lowering agricultural production. Its impact includes a 10–25% crop yield reduction and, in severe cases, desertification^6^. Salinity affects plant growth by inducing osmotic stress, ionic toxicity, and nutrient deficiencies, impacting various cellular processes and photosynthesis^1,7–9^. This stress generates reactive oxygen species (ROS), causing significant damage to plant membranes^10–12^. Although plants have developed internal defense mechanisms, such as antioxidant enzymes, some species, like wheat, remain vulnerable to salinity stress due to limitations in their defense systems^13,14^.

Gallic acid (GA), a powerful phenolic compound, benefits plants by acting as an antioxidant, protecting against oxidative stress. It promotes root growth, boosts photosynthesis, and helps plants absorb nutrients efficiently^15^. Its allelopathic properties aid in weed control, improving plant growth and health^16^. On other hand. zinc ferrite nanoparticles (ZnFNP) possess a wide range of properties beneficial for soil remediation and medical applications^17^. These nanoparticles and other ferrite variations have demonstrated potential benefits for plants by enhancing nutrient uptake efficiency^18^.

Wheat (*Triticum aestivum* L.) is the earliest domesticated cereal crop globally, cultivated by approximately 80 million farmers and contributing to the sustenance of 2.5 billion individuals worldwide^19^. Salinity stress poses a significant challenge to wheat cultivation, detrimentally impacting various physiological and biochemical processes^20^. As sodium and chloride ions accumulate in the soil, they disrupt the plant's osmotic balance, reducing water uptake and causing dehydration^21^. This environmental stress triggers a force of responses within the wheat plant, including altered ion uptake, inhibition of essential metabolic pathways, and oxidative stress due to the accumulation of reactive oxygen species (ROS)^22^.

The current study aims to investigate the potential of GA and ZnFNP to alleviate the negative effect of salinity stress on the growth of wheat plants. To mitigate salt stress on wheat, the current study covers the knowledge gap about the optimal application rate of GA in combination with ZnFNP. We hypothesized that using ZnFNP and GA would improve wheat growth and production while mitigating the negative effects of salt stress. The study's primary objective was to determine the optimal rate of GA and ZnFNP treatment for improved wheat growth in soils affected by salt.

## Material and methods

### Experimental site

**Figure 1:**
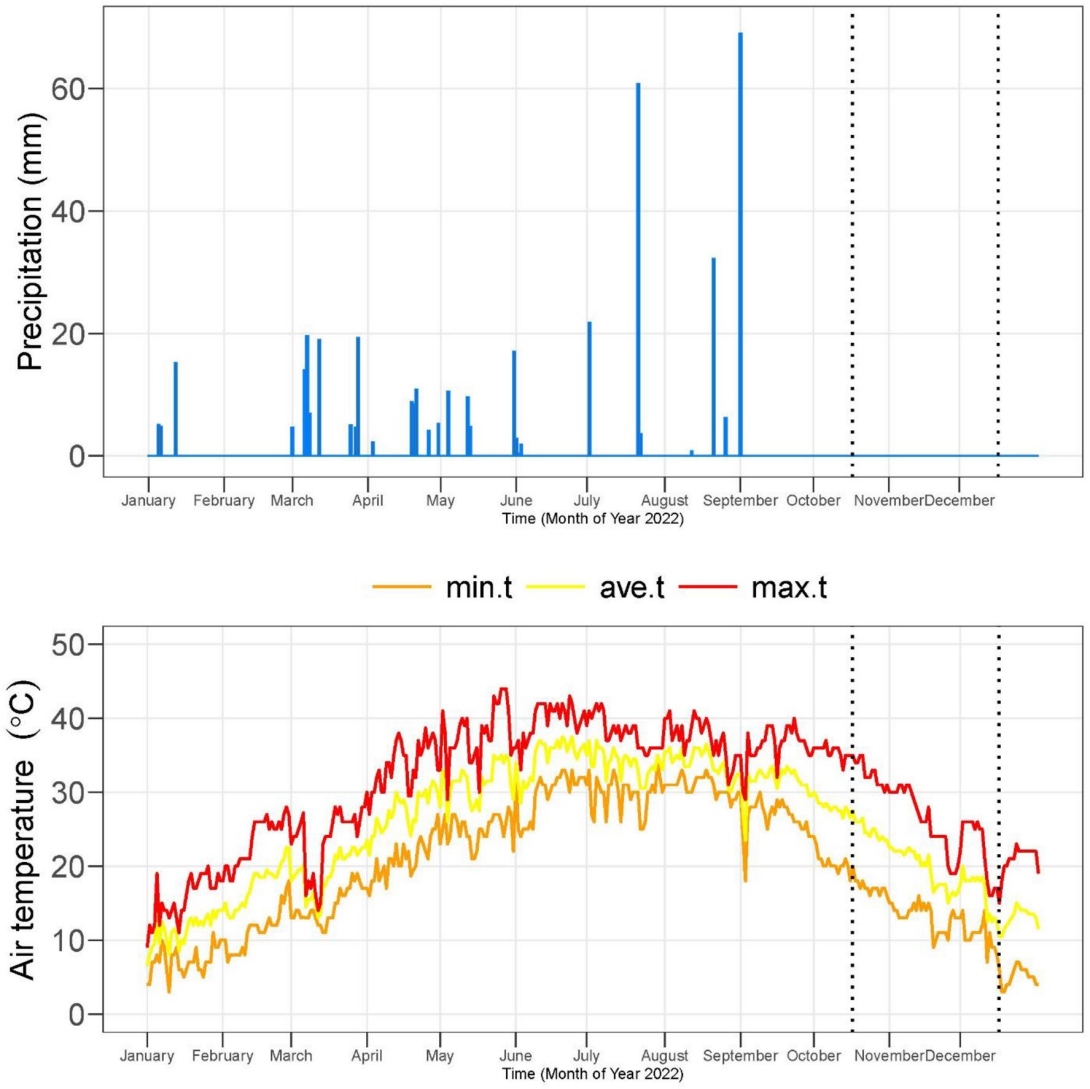
Climatic data of experiment.

In 2022, an experiment was conducted at the Research Solution experimental area (30° 09′ 41.6″ N 71° 36′ 38.0″ E). Random soil sampling was done to obtain a composite sample. These samples underwent air-drying and were sieved through a 2-mm mesh to evaluate their physicochemical properties. The physiochemical characteristics of soil and irrigation water are provided in Table 1. The climatic data of the experiment is provided in Fig. 1.

**Table 1:** Pre-experimental soil and irrigation characteristics.

Soil	Values	References	Irrigation	Values	References
pH	8.17	^23^	pH	7.20	^24^
EC*e* (dS/m)	6.40	^25^	EC (µS/cm)	381	
SOM (%)	0.55	^26^	Carbonates (meq./L)	0.02	
Extractable potassium (µg/g)	130	^27^	Sodium (mg/L)	109	
Extractable sodium (µg/g)	117	^28^	Bicarbonates (meq./L)	4.71	
Texture	Clay Loam	^29^	Ca + Mg (meq./L)	2.72	
Total nitrogen (%)	0.03	^30^	Chloride (meq./L)	0.01	
Available phosphorus (µg/g)	6.21	^31^

### Synthesis of ZnFNP

Initially, a solution containing 0.2 M Zn(NO_3_)_2_.6H_2_O and Fe(NO_3_)_3_ was prepared by dissolving them separately in deionized water under continuous stirring. Extract derived from Fenugreek (*Trigonella foenum-graecum* L.) seeds, locally referred to as Methi Dana, was obtained by washing, grinding into a paste, and subsequent filtration. The biosynthesis entailed combining the metal solution and plant extract in equal proportions (1:1 ratio) with stirring while adjusting the pH to 10 using NaOH. A noticeable color change to a dark brown or black hue indicated the emergence of ZnFe_2_O_4_ nanoparticles (NPs). Following the stirring process, the NPs were separated by centrifugation at 5000 rpm for 10–15 min. Further purification was achieved by rinsing the nanoparticles with deionized water to eliminate contaminants. The resulting ZnFe_2_O_4_ nanoparticles pellets were dried at 80 °C for 4–5 h in an oven.

### Treatments

The treatments included a control group without any treatment; levels of gallic acid (GA) include (10 µM, 15 µM, and 20 µM). All the treatments were applied under no nanoparticles (No NP) and 5 μM zinc ferrite nanoparticles (ZnFNP)^17^. A total of 2 foliar applications were made after two weeks of germination following the gap of 15 days in each foliar. The study followed a completely randomized design (CRD) with four replications for every treatment.

### Collecting, sterilization, and sowing of seeds

The Millat-2011 variety of wheat seeds was obtained from a licensed seed dealer in the local area. After manual screening, 10 seeds were planted in each pot with 5 kg of soil. To ensure that each pot included two healthy seedlings, thinning was carried out after germination.

### Fertilizer

To address the nutritional requirements of wheat, nitrogen (N) was administered at a rate of 52 kg per acre (approximately 0.31 g/10 kg soil), derived from urea, while phosphorus (P) was applied at 46 kg per acre (about 0.15 g/10 kg soil) sourced from single superphosphate, aligning with suggested application guidelines. To improve potassium (K) levels, potassium sulfate was supplemented at 25 kg per acre (around 0.15 g/10 kg soil).

### Irrigation

The regulation of irrigation for each pot was carefully managed by utilizing a moisture gauge (ADVANCED™; 4 in 1 Soil Meter; China). Daily supervision was conducted to guarantee the maintenance of moisture levels within the defined threshold, aligned with the device's scale, where the designation 'wet' represented roughly 69% of the soil's field capacity.

### Harvesting and data collection

Wheat harvesting commenced 55 days after sowing. The data collection encompassed multiple parameters such as shoot and root length, along with both fresh and dry weights of shoots and roots, conducted immediately post-harvest. The drying process for the dry weight analysis involved 48 h of oven drying at 65 °C. Chlorophyll content, electrolyte leakage, and antioxidant levels were assessed in freshly collected leaves obtained 27 days after germination. On the other hand, the concentration of N, P, and K was determined in leaves collected 55 days post-germination.

### Estimation of chlorophyll

In this study, freshly harvested wheat leaf samples weighing 0.5 mg were prepared by grinding in a mortar and pestle using 20 ml of 80% acetone. The resulting mixture was centrifuged at 3000 rpm for 15 min to obtain a supernatant. Subsequently, the residue underwent further extraction with 5 ml of 80% acetone until complete color extraction was achieved. For spectrophotometric analysis, the absorbance of specific wavelengths was measured: 663, 645, and 470 nm^32^. The determination of chlorophyll and carotenoid content was carried out using established formulae:

$${\text{Chlorophyll}}\;{\text{a}}\left( {\frac{{{\text{mg}}}}{{\text{g}}}} \right) = \frac{{\left( {13.7 \times {\text{A}}663} \right) - \left( {2.59 \times {\text{A}}645} \right) \times {\text{V}}}}{{1000 \times {\text{W}}}}$$

$${\text{Chlorophyll}}\;{\text{b}}\left( {\frac{{{\text{mg}}}}{{\text{g}}}} \right) = \frac{{\left( {20.9 \times {\text{A}}645} \right) - \left( {4.58 \times {\text{A}}663} \right) \times {\text{V}}}}{{1000 \times {\text{W}}}}$$

$${\text{Total}}\;{\text{Chlorophyll}}\left( {\frac{{{\text{mg}}}}{{\text{g}}}} \right) = \frac{{22.2\left( {{\text{A}}645} \right) + 8.05\left( {{\text{A}}663} \right) \times {\text{V}}}}{{1000 \times {\text{W}}}}$$

$${\text{Carotenoids}}\left( {\frac{{{\text{mg}}}}{{\text{g}}}} \right) = {\text{OD}}480 + 0.114({\text{OD}}\;663) - 0.638({\text{OD}}\;645)$$

### Gas exchange attributes

Leaf gas exchange parameters were evaluated using the CI-340 Photosynthesis system, an infrared gas Analyzer manufactured by CID, Inc. USA. These assessments were conducted during the peak sunlight hours from 10:30 to 11:30 a.m., ensuring an optimal and saturating light intensity for efficient photosynthesis^33^.

### Antioxidants

We evaluated superoxide dismutase (SOD) activity by using nitro blue tetrazolium and taking absorbance at 560 nm^34^. Catalase (CAT) activity was measured by observing the decomposition of hydrogen peroxide (H_2_O_2_) at 240 nm^35^. Ascorbate peroxidase (APX) activity was determined via ascorbate oxidation in the presence of H_2_O_2_ at 290 nm wavelength^36^.

### Determination of nonenzymatic antioxidants

M-phosphoric acid, Na EDTA, and sulfosalicylic acid (w/v) were used to analyze the GST content. After centrifuging the mixture at 12,000 × *g* for 10 min, the supernatant was mixed with 100 mM phosphate buffer (pH 7.0) and 5.5-dithiobis (2-nitrobenzoic acid). At 412 nm, the last absorption was measured^37^.

### Electrolyte leakage

Fresh leaf discs of 1 cm diameter were taken in 20 ml of deionized water and left to incubate at a constant temperature of 25 °C for 24 h in test tubes. After incubation, 1st electrical conductivity was measured. After that, test tubes underwent a 20-min heat treatment in a water bath at 120 °C, and the 2nd electrical conductivity measurement was recorded^38^.

$$\text{Electrolyte Leakage }\left(\%\right)=\left(\frac{\text{EC}1}{\text{EC}2}\right)\times 100$$

### Free proline determination

The procedure used to extract and analyze free proline from leaf tissues was adapted from the methodology^39^. The final absorbance was taken at 520 nm using a UV–VIS spectrophotometer, with l-proline as the calibration standard.

### Shoot and grain N, P, K

The nitrogen concentration was evaluated using a modified micro-Kjeldahl method^24^. Potassium concentrations were measured utilizing a flame photometer^27,40^. Phosphorus levels were determined at 420 nm using the yellow colorimetric technique with a spectrophotometer^24^.

### Statistical analysis

Standard statistical analysis was applied to collected data^41^. The study utilized OriginPro software for two-way ANOVA, paired comparisons, graph generation, and principal component analysis^42^.

### Ethics approval and consent to participate

We all declare that manuscript reporting studies do not involve any human participants, human data, or human tissue. So, it is not applicable.

### Experimental research and field studies on plants (either cultivated or wild), including the collection of plant material, must comply with relevant institutional, national, and international guidelines and legislation

We confirmed that all methods were performed in accordance with the relevant guidelines/regulations/legislation.

## Results

### Shoot attributes

**Figure 2:**
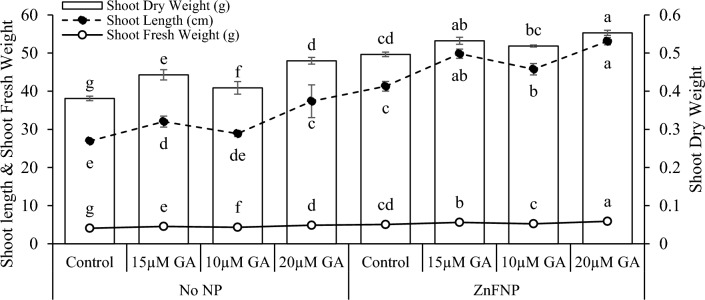
Effect of different gallic acid (GA) levels with and without ZnFNP on shoot length, shoot fresh and dry weight of wheat grown with no NP and ZnFNP. The bars showed the mean of 4 replicates with ± SD. Significant changes, denoted by distinct letters on the bars, were identified through the Tukey test at a significance level of p < 0.05.

In no NP, applying 15 µM GA resulted in an 18.92% increase, while 10 µM GA showed a 7.27% increase, and 20 µM GA exhibited 38.77% rise in shoot length over control. With ZnFNP, 15 µM GA treatment showed a 20.68% increase in shoot length than the ZnFNP control. Treatment 10 µM GA demonstrated a 10.85% enhance, while 20 µM GA showed the highest increase of 28.62% in shoot length from ZnFNP control (Fig. 2).

At no NP, treatment 15 µM GA resulted in an 11.60% increase, while 10 µM GA showed a 6.08% increase, and 20 µM GA exhibited the most significant rise at 19.34% in shoot fresh weight then the control. Under ZnFNP applying 15 µM GA led to a 10.98%, 10 µM GA showed a 3.66%, and 20 µM GA showed a 16.52% increase in shoot fresh weight in comparison to control (Fig. 2).

Results showed that 15 µM GA treatment showed a 16.27%, while the 10 µM GA displayed a 7.28% rise in shoot dry weight above the control. Over the control, adding 20 µM GA treatment resulted in an increase of 25.92% in shoot dry weight. Treatment 15 µM GA + ZnFNP showed a 7.20%, 10 µM GA displayed a 4.43%, and 20 µM GA caused in an 11.38% improve shoot dry weight related to the control (Fig. 2).

### Root attributes

**Figure 3:**
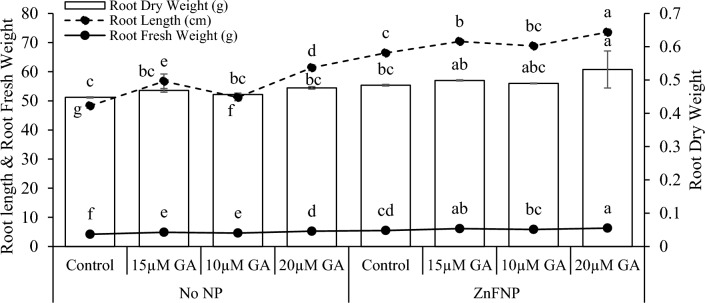
Effect of different gallic acid (GA) levels with and without ZnFNP on root length, root fresh and dry weight of wheat grown with no NP and ZnFNP. The bars showed the mean of 4 replicates with ± SD. Significant changes, denoted by distinct letters on the bars, were identified through the Tukey test at a significance level of p < 0.05.

Adding 15 µM GA, 10µ GA, and 20 µM GA with no NP resulted in an increase in root length (17.46%, 27.15%, and 6.04%), root fresh weight (16.04%, 9.84%, and 25.04%), and root dry weight (4.66%, 1.91%, and 6.38%) from the control. When 15 µM GA, 10µ GA, and 20 µM GA treatments applied with ZnFNP resulted a significant increase in root length (5.97%, 10.76%, and 3.64%), root fresh weight (11.48%, 6.42%, and 14.72%), and root dry weight (2.94%, 1.10%, and 9.71%) over the control (Fig. 3).

### Chlorophyll and carotenoid content

**Figure 4:**
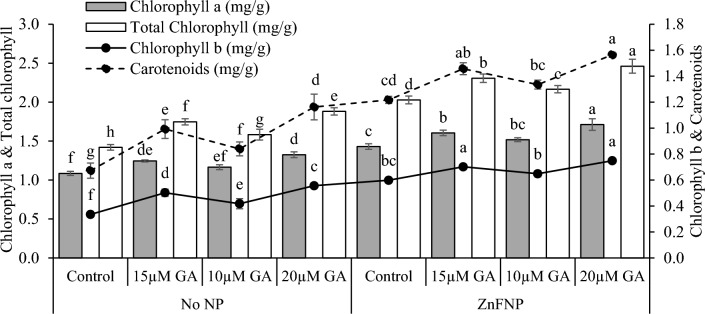
Effect of different gallic acid (GA) levels with and without ZnFNP on chlorophyll, chlorophyll b, total chlorophyll, and carotenoids of wheat grown with no NP and ZnFNP. The bars showed the mean of 4 replicates with ± SD. Significant changes, denoted by distinct letters on the bars, were identified through the Tukey test at a significance level of p < 0.05.

In the no NP, the application of 15 µM GA, 10µ GA, and 20 µM GA resulted in an increase in photosynthetic rate (17.56%, 9.44%, and 30.87%), transpiration rate (14.56%, 8.04% and 18.86%), and stomatal conductance (15.19%, 7.81%, and 29.07%) than the control. Applying 15 µM GA, 10µ GA, and 20 µM GA treatments with ZnFNP resulted in a significant increase in photosynthetic rate (9.07%, 4.89%, and 12.72%), transpiration rate (6.84%, 3.59%, and 10.09%), and stomatal conductance (11.84%, 6.62%, and 15.25%) in comparison to the control (Fig. 4).

### Gass exchange attributes

**Figure 5:**
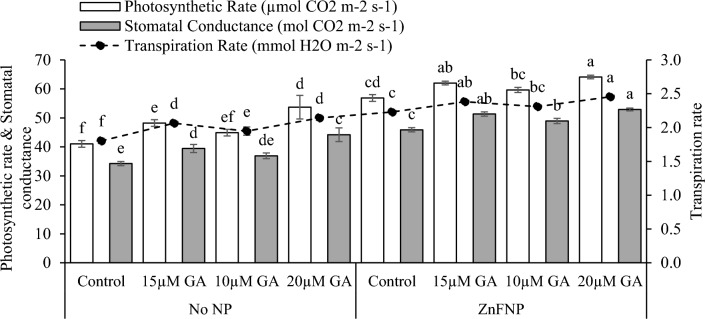
Effect of different gallic acid (GA) levels with and without ZnFNP on photosynthetic rate, transpiration rate, and stomatal conductance of wheat grown with no NP and ZnFNP. The bars showed the mean of 4 replicates with ± SD. Significant changes, denoted by distinct letters on the bars, were identified through the Tukey test at a significance level of p < 0.05.

In the no NP, the application of 15 µM GA, 10µ GA, and 20 µM GA showed in rise in photosynthetic rate (17.56%, 9.44%, and 30.87%), transpiration rate (14.56%, 8.04% and 18.86%), and stomatal conductance (15.19%, 7.81%, and 29.07%) than the control. Applying 15 µM GA, 10µ GA, and 20 µM GA treatments with ZnFNP resulted in a significant increase in photosynthetic rate (9.07%, 4.89%, and 12.72%), transpiration rate (6.84%, 3.59%, and 10.09%), and stomatal conductance (11.84%, 6.62%, and 15.25%) in comparison to the control (Fig. 5).

### Electrolyte leakage, proline, SOD, and CAT activity

**Figure 6:**
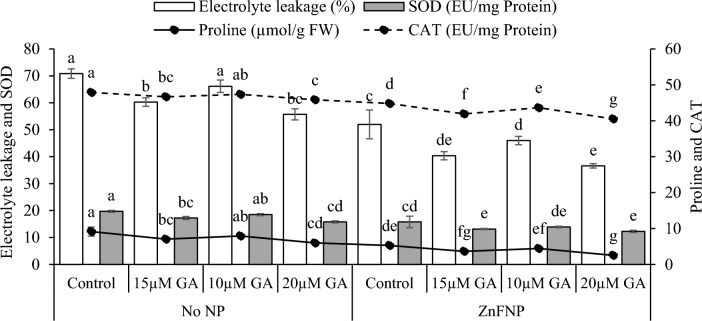
Effect of different gallic acid (GA) levels with and without ZnFNP on electrolyte leakage, proline, SOD, and CAT of wheat grown with no NP and ZnFNP. The bars showed the mean of 4 replicates with ± SD. Significant changes, denoted by distinct letters on the bars, were identified through the Tukey test at a significance level of p < 0.05.

Adding 15 µM GA, 10µ GA, and 20 µM GA showed a significant decrease in electrolyte leakage (17.55%, 7.11%, and 27.13%), proline (29.95%, 15.36%, and 52.86%), SOD (14.31%, 6.61%, and 24.94%), and CAT (2.63%, 1.14%, and 4.45%) than the control under no NP. Adding 15 µM GA, 10 µ GA, and 20 µM GA treatments resulted a significant decrease in electrolyte leakage (28.85%, 13.01%, and 42.07%), proline (45.06%, 19.29%, and 108.79%), SOD (20.11%, 13.44%, and 28.63%), and CAT (6.91%, 2.66%, and 10.58%) over the control under ZnFNP (Fig. 6).

### APX, GPX, GR, and GST activity

**Figure 7:**
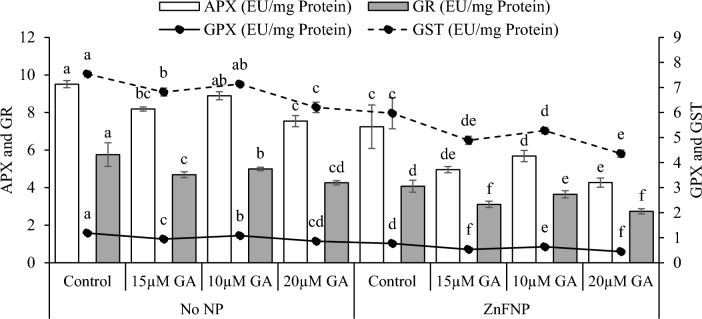
Effect of different gallic acid (GA) levels with and without ZnFNP on APX, GPX, GR, and GST of wheat grown with No NP and ZnFNP. The bars showed the mean of 4 replicates with ± SD. Significant changes, denoted by distinct letters on the bars, were identified through the Tukey test at a significance level of p < 0.05.

In comparison to the control, applying 15 µM GA, 10µ GA, and 20 µM GA showed a significant decrease in APX (16.15%, 6.94%, and 26.09%), GPX (25.40%, 9.47%, and 38.19%), GR (22.88%, 15.32%, and 35.29%), and GST (10.56%, 5.64%, and 21.47%) under no NP. Applying 15 µM GA, 10µ GA, and 20 µM GA treatments exhibited a significant decrease in APX (46.09%, 27.45%, and 69.71%), GPX (44.60%, 21.26%, and 73.03%), GR (31.13%, 11.80%, and 48.72%), and GST (22.09%, 13.18%, and 37.01%) from the control under ZnFNP (Fig. 7).

### Shoot N, P, and K

Adding 15 µM GA, 10µ GA, and 20 µM GA with no NP increased shoot N (54.93%, 25.35%, and 83.10%), shoot P (55.58%, 22.51%, and 76.49%), and shoot K (85.23%, 34.23%, and 99.40%) than the control. When 15 µM GA, 10µ GA, and 20 µM GA treatments were applied with ZnFNP exhibit a significant rise in shoot N (25.41%, 12.54%, and 45.21%), shoot P (21.45%, 12.41%, and 28.70%), and shoot K (25.56%, 12.92%, and 40.45%) over the control (Table 2).

**Table 2:** Effect of different gallic acid (GA) levels with and without ZnFNP on wheat shoot N, P and K concentration.

Treatment	Shoot N (%)	Shoot P (%)	Shoot K (%)
No NP			
Control	0.36g	0.13f	0.37g
15 µM GA	0.55ef	0.20d	0.69e
10 µM GA	0.45fg	0.15e	0.50f
20 µM GA	0.65de	0.22d	0.78de
ZnFNP			
Control	0.76cd	0.25c	0.89cd
15 µM GA	0.95b	0.31ab	1.12b
10 µM GA	0.85bc	0.28b	1.01bc
20 µM GA	1.10a	0.32a	1.25a

Values are the mean of 4 replicates. Different letters showed significant changes at p ≤ 0.05; Tukey Test. No NP (No Nanoparticle); ZnFNP (Zinc ferrite Nanoparticle).

### Grain N, P, and K

Over control, adding 15 µM GA, 10µ GA, and 20 µM GA with no NP, exhibits an increase in grain N (1.77%, 3.53%, and 1.69%), grain P (13.91%, 6.20%, and 23.40%), and grain K (10.87%, 5.63%, and 15.95%) in comparison to the control. When 15 µM GA, 10µ GA, and 20 µM GA treatments were applied with ZnFNP, showed a significant increase in grain N (5.08%, 3.44%, and 1.85%), grain P (9.77%, 6.70%, and 19.68%), and grain K (10.87%, 6.11%, 17.23%) over the control (Table 3).

**Table 3:** Effect of different gallic acid (GA) levels with and without ZnFNP on wheat grains N, P and K concertation.

Treatment	Grain N (%)	Grain P (%)	Grain K (%)
No NP			
Control	1.77e	0.27f	0.46f
15 µM GA	1.84de	0.30de	0.51de
10 µM GA	1.80de	0.28ef	0.48ef
20 µM GA	1.86cd	0.33cd	0.53d
ZnFNP			
Control	1.89bcd	0.36bc	0.54cd
15 µM GA	1.96ab	0.39b	0.60b
10 µM GA	1.93bc	0.38b	0.58bc
20 µM GA	2.03a	0.43a	0.64a

Values are the mean of 4 replicates. Different letters showed significant changes at p ≤ 0.05; Tukey Test. No NP (No Nanoparticle); ZnFNP (Zinc ferrite Nanoparticle).

### Convex hull and Hierarchical cluster analysis

**Figure 8:**
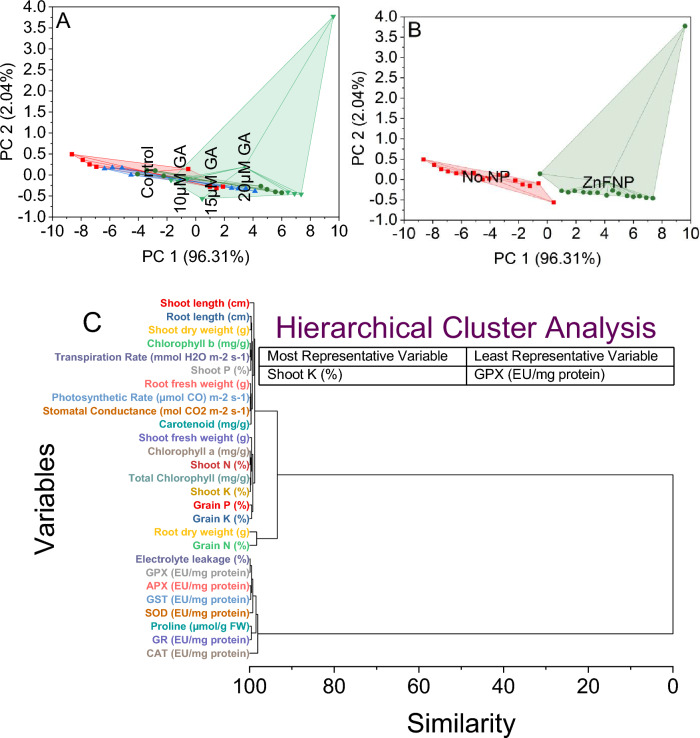
Cluster plot convex hull for treatments (**A**), NP levels (**B**), and hierarchical cluster plot (**C**) for studied attributes.

The analysis was based on PC1 and PC2 scores corresponding to different treatments. In the control group, PC1 scored between − 8.63927 and 1.90773, while PC2 ranged from − 0.56659 to 0.49291. Treatment with 15 µM GA showed a shift in the convex hull with PC1 scores ranging from − 4.0616 to 5.99969 and PC2 from − 0.42279 to 0.11149. Similarly, the 10 µM GA treatment displayed a distinct pattern, with PC1 scores varying between − 6.36301 and 4.13899 and PC2 between − 0.41066 and 0.17451. The highest variation was observed with the 20 µM GA treatment, spanning from − 1.7232 to 9.60124 for PC1 and − 0.56659 to 3.77421 for PC2 (Fig. 8A).

For the stress condition without nanoparticles (No NP), the convex hull covered a cluster of points with PC1 scores ranging from − 8.63927 to 0.45056 and PC2 scores between − 0.56659 and 0.49291. Within this grouping, the stress condition labeled as ZnFNP exhibited a distinct set of points forming another convex hull, ranging from − 0.52107 to 9.60124 in PC1 and from − 0.4586 to 3.77421 in PC2. The convex hull analysis outlines the spatial distribution of data points, showing a clear separation between stress conditions, particularly the differentiation between No NP and ZnFNP based on their PC1 and PC2 scores (Fig. 8B).

For instance, electrolyte leakage and GPX shared a similarity score of 0.12434, indicating their proximity in response patterns. Chlorophyll a and shoot N displayed a similarity score of 0.13838, suggesting a close relationship between these parameters. Variables like chlorophyll b and transpiration rate, photosynthetic rate, and stomatal conductance exhibited a slightly higher similarity score of 0.18045 and 0.18059, respectively, indicating their comparable responses. As the similarity scores increased, the clustering showed relationships between root length and shoot dry weight with a score of 0.34492, denoting their closer association. Variables such as shoot length and shoot fresh weight had a similarity score of 1.01124, indicating their stronger association than other variables. Variables like SOD and CAT displayed higher similarity scores of 0.76518 and 1.9309, respectively, potentially indicating a distinct response pattern from other variables within this analysis. Variables such as grain N and root dry weight exhibited a much higher similarity score of 1.66212 (Fig. 8C).

### Pearson correlation analysis

**Figure 9:**
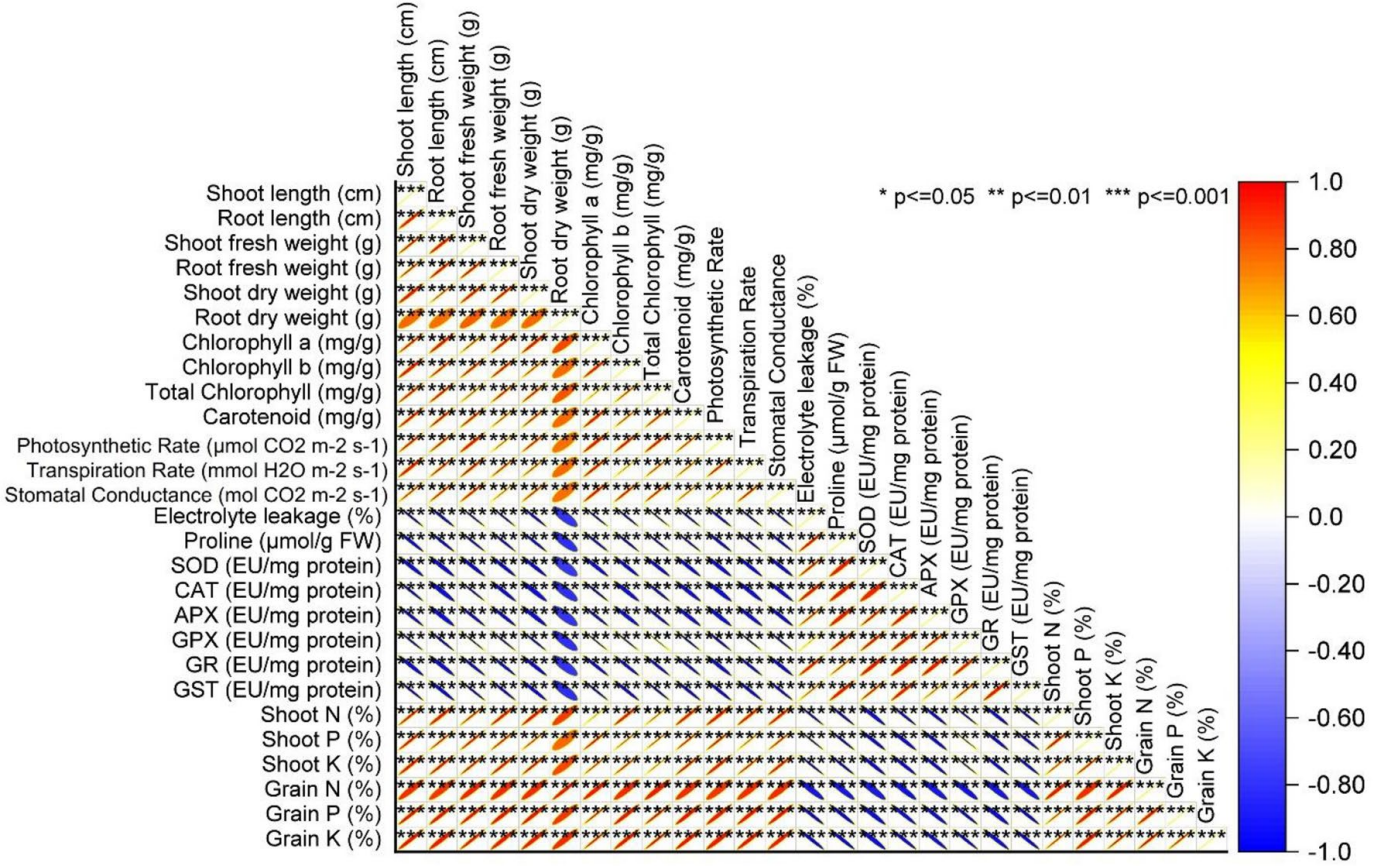
Pearson correlation analysis of the studied attributes.

Plant growth-related metrics, such as shoot length demonstrated a strong positive correlation with root length at 0.9751, while biomass indicators, like shoot fresh weight and root fresh weight, exhibited a robust correlation of 0.98674. Similarly, parameters reflecting pigment content, including chlorophyll a and chlorophyll b, displayed a highly correlated relationship of 0.98658. Notably, physiological traits connected to photosynthesis and transpiration, such as photosynthetic and transpiration rates, also depicted strong positive correlations around 0.98628. However, stress-related markers, notably electrolyte leakage, exhibited a negative correlation ranging from − 0.95411 to − 0.99533 with key antioxidant enzyme activities (SOD, CAT, APX, GPX, GR, GST). Conversely, parameters linked to nutrient content, like shoot N, shoot P, shoot K, grain N, grain P, and grain K, demonstrated consistent positive correlations between 0.8984 and 0.99729, suggesting interdependence among these nutritional factors (Fig. 9).

**Figure 10:**
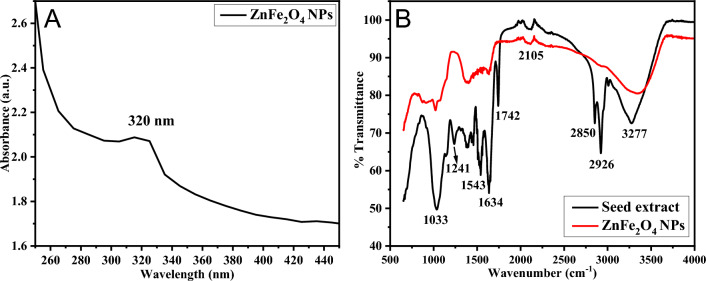
UV–Vis absorption spectrum of *Trigonella foenum-graecum* L. seeds extract-stabilized ZnFe_2_O_4_ NPs NPs (**A**). FTIR spectra of pure *Trigonella foenum-graecum* L. seeds powder and the ligand-stabilized ZnFe_2_O_4_ NPs (**B**).

Figure 10A displays an absorption band centered around 320 nm in the Fenugreek seed extract stabilized ZnFe_2_O_4_ NPs. Within the spectrum, the 1033 cm^−1^ absorption band suggests the presence of C–O (alcohols, ethers, carboxylic acid, etc.), C–N (amines), and C–F vibrations from the extract. This band appears to facilitate the reduction of metal ions to zero valent NPs while simultaneously stabilizing them to prevent environmental oxidation. Another significant 1241 cm^−1^ absorption band, attributed to C–N amine, was evident in the Fenugreek seed extract but absent in the ZnFe_2_O_4_ NPs spectrum. Weak signals in the 1387–1460 cm^−1^ range were identified as bending vibrations of C–H alkanes, while sharper bands around 1543 cm^−1^ were linked to N–H bending in amide II. These IR vibrations were discernible, albeit with weak signals, in the functionalized NPs sample. The 1634 cm^−1^ absorption band, recognized as amide I, confirms the presence of amide linkages in proteins within the NPs sample, indicating the presence of carbonyl functional groups. Additionally, a moderate intensity C=O stretching bond observed at 1742 cm^−1^ suggests the presence of lipids, such as esters, in the extract but was absent in the Fenugreek seed extract stabilized NPs sample. An IR peak around 2105 cm^−1^ indicates the C≡C alkyne vibration observed in both samples (Fig. 10B). Furthermore, the absence of absorption bands at 2850 and 2926 cm^−1^, attributed to the stretching vibrations of symmetric and asymmetric SP3 C–H, in the NPs surface-bound Fenugreek seed ligand might suggest a non-covalent C-H binding to the NPs surfaces. In the FTIR analysis of *Trigonella foenum-graecum* L. extract, an absorption band at 3277 cm^−1^ was identified, indicating N–H stretching vibrations and O–H bonds formed from essential amides and starch in the green ligand.

## Discussion

Salinity stress significantly impacts crops, disrupting their water balance and nutrient uptake, reducing growth, yield, and overall plant health^43^. It increases osmotic stress, hindering the plant's ability to absorb water, and ultimately affecting crop productivity and quality^44^. Salinity-induced stress induces oxidative stress in plants. Elevated salt concentrations prompt the production of reactive oxygen species (ROS) within plant cells. These ROS, including hydrogen peroxide (H_2_O_2_) and superoxide radicals (O^2^), inflict oxidative harm on cellular structures like lipids, proteins, and DNA. Consequently, this oxidative stress disturbs typical cellular processes and may ultimately result in cellular demise^45,46^. A significant decline in the growth of wheat also validated above arguments especially in control when wheat was cultivated in salinity stress.

Gallic acid (GA) enhances plant growth by stimulating cell elongation, aiding in photosynthesis and nutrient absorption, and acting as a potent growth regulator with antioxidant properties^47^. It also remediates the growth via improvement in water absorption capability which minimize the salinity induced osmotic stress^48,49^. Furthermore, in salt-treated plants, there was an increase in phenolic compounds, such as total phenols and flavonoids, due to GA. These compounds could potentially aid in scavenging excess reactive oxygen species (ROS) produced under such conditions^50,51^. GA exhibits potential antioxidant properties by mitigating lipid peroxidation and oxidative damage within plants. It achieves this by scavenging ROS and enhancing the antioxidant defense system and metabolic processes. This action helps to maintain cellular integrity and protect the plant against oxidative stress-induced harm^52^. Application of 20 µM GA + 5 µM ZnFNP demonstrated similar improvements in minimization of oxidative stress due to modulation in the antioxidants i.e., POD, SOD, APX etc.

Furthermore, ZnFe_2_O_4_ NPs have antioxidant qualities that help plants stressed by salt by efficiently scavenging reactive oxygen species (ROS) and boosting the activity of antioxidant enzymes as superoxide dismutase (SOD), catalase (CAT), and peroxidase (POD)^17^. Furthermore, the application of ZnFe_2_O_4_ NPs stimulates the synthesis of non-enzymatic antioxidants, including phenolic compounds and flavonoids, thereby bolstering the overall antioxidant defense system of plants. Collectively, these mechanisms contribute to the alleviation of oxidative damage and enhancement of plant resilience to salinity stress, ultimately promoting growth and productivity in affected crops^17^. In addition to above, it also has potential to enhance the uptake of nutrients in the plants that play an integral part in improvement of plant growth under stress conditions^18^. Similar kind of results were also noted, in current study where grains and shoot N, P and K were significantly enhanced over control where 20 µM GA + 5 µM ZnFNP was applied as treatment.

## Conclusion

In conclusion, 20 µM GA + 5 µM ZnFNP can enhance wheat growth under salinity stress. Applying 20 µM GA + 5 µM ZnFNP also can also potentially improve N, P, and K in both grain and shoot of wheat grown in salinity stress. Moreover, the 20 µM GA + 5 µM ZnFNP treatment can regulate antioxidant levels in salinity, which might decrease the negative impacts of salinity on wheat. Further field studies are suggested for the declaration of 20 µM GA + 5 µM ZnFNP as a potential amendment for alleviating salinity stress in wheat.

## Data Availability

All data generated or analyzed during this study are included in this published article.
